# Computational Modeling of Therapy with the NMDA Antagonist in Neurodegenerative Disease: Information Theory in the Mechanism of Action of Memantine

**DOI:** 10.3390/ijerph19084727

**Published:** 2022-04-14

**Authors:** Dariusz Świetlik, Aida Kusiak, Agata Ossowska

**Affiliations:** 1 Division of Biostatistics and Neural Networks, Medical University of Gdańsk, Dębinki 1, 80-211 Gdańsk, Poland; 2 Department of Periodontology and Oral Mucosa Diseases, Medical University of Gdańsk, 80-204 Gdańsk, Poland; akusiak@gumed.edu.pl (A.K.); agata.ossa@wp.pl (A.O.)

**Keywords:** NMDA antagonists, memantine, Alzheimer’s disease, neural networks, computer simulation, virtual therapy

## Abstract

(1) Background: in patients with neurodegenerative diseases, noncompetitive N-methyl-D-aspartate (NMDA) receptor antagonists provide neuroprotective advantages. We performed memantine therapy and proved mathematical and computer modeling of neurodegenerative disease in this study. (2) Methods: a computer simulation environment of the N-methyl-D-aspartate receptor incorporating biological mechanisms of channel activation by high extracellular glutamic acid concentration. In comparison to controls, pathological models were essentially treated with doses of memantine 3–30 µM. (3) Results: the mean values and 95% CI for Shannon entropy in Alzheimer’s disease (AD) and memantine treatment models were 1.760 (95% CI, 1.704–1.818) vs. 2.385 (95% CI, 2.280–2.490). The Shannon entropy was significantly higher in the memantine treatment model relative to AD model (*p* = 0.0162). The mean values and 95% CI for the positive Lyapunov exponent in AD and memantine treatment models were 0.125 (95% CI, NE–NE) vs. 0.058 (95% CI, 0.044–0.073). The positive Lyapunov exponent was significantly higher in the AD model relative to the memantine treatment model (*p* = 0.0091). The mean values and 95% CI for transfer entropy in AD and memantine treatment models were 0.081 (95% CI, 0.048–0.114) vs. 0.040 (95% CI, 0.019–0.062). The transfer entropy was significantly higher in the AD model relative to the memantine treatment model (*p* = 0.0146). A correlation analysis showed positive and statistically significant correlations of the memantine concentrations and the positive Lyapunov exponent (correlation coefficient R = 0.87, *p* = 0.0023) and transfer entropy (TE) (correlation coefficient R = 0.99, *p* < 0.000001). (4) Conclusions: information theory results of simulation studies show that the NMDA antagonist, memantine, causes neuroprotective benefits in patients with AD. Our simulation study opens up remarkable new scenarios in which a medical product, drug, or device, can be developed and tested for efficacy based on parameters of information theory.

## 1. Introduction

Dementia currently affects roughly 47 million people globally, with forecasts indicating that it will affect almost 80 million people in the next ten years, with Alzheimer’s disease (AD) being the most frequent cause [1,2]. Alzheimer’s disease will affect 65 million people in 2030 and 115 million in 2050, according to the World Health Organization (WHO). By 2050, the number of sufferers in Poland will have tripled to almost 1 million [2]. Memory and other cognitive processes are among the clinical indications of Alzheimer’s disease, which is a rapidly progressing neurodegenerative illness [3,4,5,6,7,8]. Most neurotransmitter systems in the brains of Alzheimer’s patients display transmission abnormalities, with glutamatergic dysfunction being the most apparent. Glutamate plays a key role in learning and memory trace development, particularly in the mechanism of the so-called long-term potentiation (LTP) phenomenon [9,10].

Memantine has the best pharmacological profile and tolerability when compared to noncompetitive NMDA receptor antagonists in preclinical research and clinical trials [11,12,13,14,15]. Memantine has been found to improve cognitive capacities and reduce neurodegeneration in Alzheimer’s disease patients [15,16].

Positive Lyapunov exponents, correlative dimensions, Shannon entropy, entropy transfer, and mutual information are among the methods used to describe the complexity of biological systems [17,18,19,20]. The utility of entropy in the interpretation of electroencephalography (EEG) signals has been proven in numerous investigations of patients with Alzheimer’s disease [21,22,23,24,25].

Due to the limitations of modern research methods, we cannot examine the nervous system in natural conditions. Computer models of neurons [26] and neural networks [27,28] are two methods for comprehending the nervous system’s functioning. Understanding the process of neurodegeneration in Alzheimer’s disease is aided by computer models of synaptic degradation in the hippocampus for various stages of synaptic loss [29,30]. Other simulation experiments, on the other hand, demonstrate that generating gamma oscillations in the hippocampus can help with the pathophysiology of Alzheimer’s disease [31]. Artificial neural networks have been successfully applied in nuclear medicine in the detection of Alzheimer’s disease based on cerebral perfusion single-photon emission computed tomography (SPECT) data [32,33] and dentistry [34]. We are the first to present a mathematical model of the NMDA receptor that allows simulation of excitotoxicity and virtual memantine therapy [34]. We showed that the NMDA antagonist, memantine, causes neuroprotective benefits in patients with moderate to severe AD.

Using the mathematical framework from earlier simulation research [26,27,28,29,30,31,35], a computer simulation environment of α-amino-3-hydroxy-5-methyl-4-isoxazolepropionic (AMPA) and NMDA receptors was established for therapy with the NMDA receptor antagonist—memantine. (1) the biological mechanism of AMPA and NMDA receptor function, (2) simulations of glutamate release inside the synaptic gap following presynaptic stimulation, (3) the mechanism of excitotoxicity, and (4) simulations of memantine treatments at three concentrations: 3, 10, and 30 µM, due to the pattern of long-term potentiation. The findings of the in silico virtual therapy study point to new bioinformatics possibilities, such as simulating real biological processes in a virtual environment.

In Section 2, we introduce mathematical models: Section 2.1 study design—mathematical model of synaptic properties, Section 2.2 neurodegenerative model—AD, Section 2.3 therapy with the NMDA antagonist memantine. In simulations comparing the control model with pathological models and virtual therapy results of memantine, the following parameters were used: parameters in a complex system—neurodegenerative disease Section 2.4, parameters of information theory Section 2.5, and parameters of synaptic transmission Section 2.6. Additionally, in Section 2.7, we describe a statistical analysis. In Section 3, we present our results, parameters in a complex system—neurodegenerative disease Section 3.1, information theory Section 3.2, and synaptic transmission Section 3.3, while relationships between memantine concentrations and parameters in a complex system and parameters of information theory, synaptic transmission, are presented in Section 3.4 and Section 3.5. In Section 4, we fully discuss our results, while Section 5 summarizes the conclusions. In Section 6, we present future directions in computer simulation neurodegenerative diseases, such as Alzheimer’s. In Section 7, we discuss study limitations.

In order to investigate information theory in the mechanism of action of memantine, we conducted a computer simulation of therapy with an NMDA antagonist.

## 2. Materials and Methods

**Figure 1:**
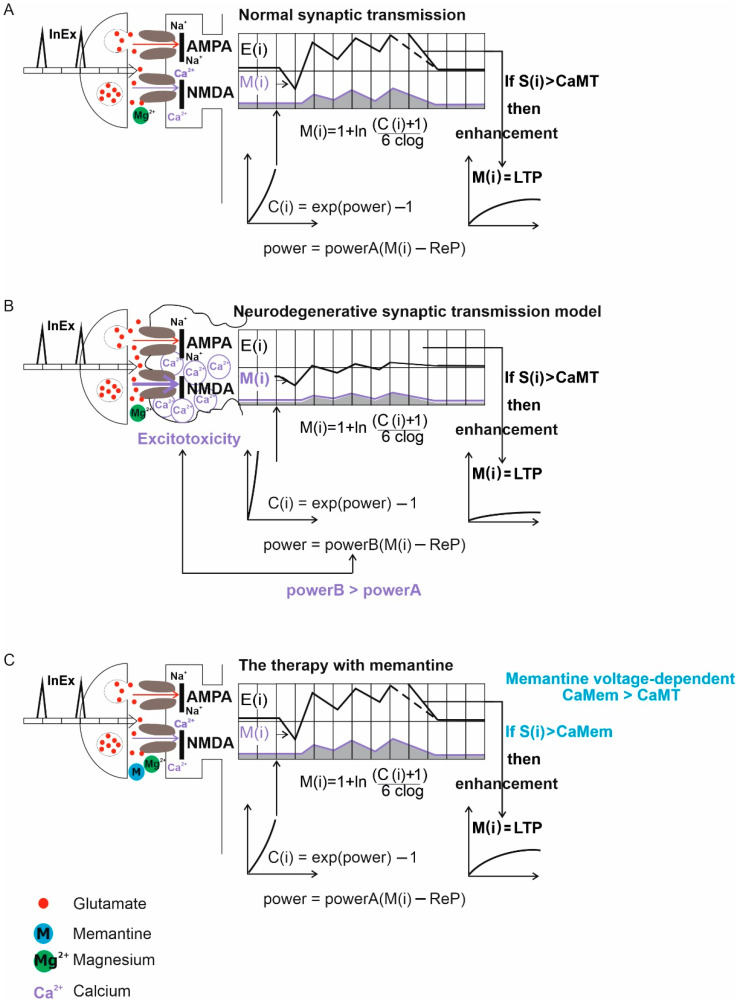
NMDA receptor activity in physiological (normal) synaptic transmission and pathological (neurodegenerative) dementia synaptic transmission situations, as well as treatment with memantine. (**A**) Normal synaptic transmission. The unblocking of the channel and the influx of Ca^2+^ ions into the cell are caused by the stimulus-induced activation of the receptor. After the arrival of an action potential, the input function InEx for excitatory synapses adds the values of the synaptic function to the relevant tables of shift registers: E(i)—is the tables of shift registers for excitatory inputs (glutamate receptors): AMPA and M(i)—is the tables of shift registers for excitatory inputs (glutamate receptors): NMDA. S(i)—is the actual value of summarized potential and, if S(i) > CaMT (CaMT = –68 mV—threshold for the removal of the Mg ion block for NMDA channels) then improvements will be made LTP. C(i)—LTP time, ReP = −80 mV (resting potential value), powerA, clog parameters. (**B**) Neurodegenerative synaptic transmission model. When neurotoxic substances activate the receptor, Mg^2+^ is released and an uncontrolled influx of Ca^2+^ into the cell occurs. Overactivation of glutamate (excitotoxicity) causes neuronal injury, and overactivation causes an increase in energy demand. The “powerB” (powerB > powerA) parameter is gradually increased to model the increase in extracellular glutamate concentration caused by over-stimulation of NMDA receptors and aggravation of excitotoxicity. The following values were used in the control model and the strength of the excitotoxicity phenomenon, respectively: 9, 56.7, 63, and 135. (**C**) The therapy with memantine. The depolarization induced by a strong stimulation is enough to break the blockage of the memantine channel and allow calcium ions to flow into the cell. NMDA receptor currents are inhibited by memantine in a concentration-dependent manner. Changes in the threshold for removing the Mg^2+^ ion block for NMDA channels were used to imitate virtual treatment (CaMem > CaMT). The following values were used in the control model and therapy with memantine, respectively: −68, −65, −63, and −55 mV.

The individual components that made up the computer simulation model will be described in detail in this section. Each module was in charge of a certain aspect of the synaptic transmission process. Formalism from previous research was used in the simulation model [26,27,28,29,30,31,35]. Each module was in charge of a certain aspect of the synaptic transmission process. The program code is available on the website: https://github.com/dswietlik/Dariusz-Swietlik/blob/main/Neuron%20model (accessed on 17 February 2022). Mathematical modeling of normal, neurodegenerative synaptic transmission, and therapy with memantine are shown in Figure 1 and use the formalism from previous studies [35].

### 2.1. Study Design—Mathematical Model of Synaptic Properties

The simulation model is based on shift register tables, with each table having synaptic inputs associated with it. The excitatory synaptic inputs have two registers, which correspond to the glutamine receptor AMPA and to the glutamine receptor NMDA. According to the two registers, E(*t*) and M(*t*), the synaptic function SF(*t*) comes in two forms, SF_AMPA_(*t*) and SF_NMDA_(*t*) [28]. All values in tables in the registers are filled with the residual potential value ReP = −80 mV at the start of each simulation Equation (1). The synaptic function sends the calculated values into the associated register when an action potential arrives at a particular input.

(1) $$\mathrm{SF}\left(t\right)=\{\begin{array}{cc} 0,\; & t=t_{\mathrm{sd}}\\ \frac{A_{\mathrm{MAX}}}{t_{r}}\left(t-t_{\mathrm{sd}}\right), & t_{\mathrm{sd}}<t\leq t_{r}\\ \frac{A_{\mathrm{MAX}}}{t_{d}}\left[\left(t_{d}-\left(t-\left(t_{r}+t_{\mathrm{sd}}\right)\right)\right)\right], & t_{r}<t\leq t_{d} \end{array},$$

where: *t*_sd_—time of synaptic delay, *t_r_*—time of EPSP/IPSP rise, *t_d_*—time of EPSP/IPSP decay, 1 millisecond (ms) = 2 steps of i. Parameters for EPSP_AMPA_: *A*_MAX_ = 5 mV, *t*_sd_ = 1 ms, *t_r_* = 2 ms, *t_d_* = 13 ms, for EPSP_NMDA_: *A*_MAX_ = 1 mV, *t*_sd_ = 1 ms, *t_r_* = 2 ms, *t_d_* = 13 ms, and for IPSP_GABA_: *A*_MAX_ = −2.5 mV, *t*_sd_ = 1 ms, *t_r_* = 2 ms, *t_d_* = 10 ms.

The synaptic membrane potential controls the activation of NMDA receptors and the opening of ion channels. Magnesium ions (Mg^2+^) from the extracellular space enter the channel at resting membrane potential and momentarily block the passage of calcium ions (Ca^2+^) and sodium ions (Na^+^) by closing the channel’s lumen. If postsynaptic receptors are significantly excited by glutamic acid at the same time, and the overall potential is greater than the threshold for opening the NMDA channel for calcium ions (−68 mV), the unblocked channel becomes permeable to Na^+^ and Ca^2+^ ions, which infiltrate the cell and stimulate it. In most circumstances, preceding activation of AMPA receptors, which mediate ion transport into the cell, is required to activate the NMDA receptor. The calcium ions entering through potential-activated NMDA receptor channels are thought to be the driving force behind the creation of synaptic plasticity, which is important to cognitive processes. As a result, physiological NMDA receptor stimulation is required for neuroplasticity and LTP processes [36].

### 2.2. Long-Term Potentiation (LTP)

Modeling the plasticity of biological synapses is difficult, but our algorithm closely resembles the biologically important process of long-term potentiation (LTP) while also accounting for forgetting, which is the return of the weight of a given synapse to its initial state in the absence of maintenance mechanisms [26,27,28,29,30,31]. LTP induction happens when there is an action potential on excitatory input and open NMDA channels due to adequate depolarization of the postsynaptic region, Figure 1.

(2) $$M\left(i\right)=1+\ln\frac{\left(C\left(i\right)+1\right)}{6\;\mathrm{clog}},$$

where CaMT = −68 mV (threshold for the removal of the Mg^2+^ ion block for NMDA channels), C(i) time of memory, clog parameter = 2.3026.

We modeled that phenomenological event by the power function:

power = powerA (M − ReP),(3)

where powerA = 9 is a parameter and M is the actual value of the synaptic function SF(*t*) for excitatory postsynaptic potentials.

### 2.3. Neurodegenerative Model—AD

Excessively high glutamate concentrations can cause NMDA receptor stimulation and a large influx of Ca^2+^ ions into the cell [36]. The amount of Ca^2+^ calcium ions entering the cell through open NMDA channels determines the start of the cascade of metabolic processes that lead to LTP. The “power” function is used to model this process. Glutamate overactivity (excitotoxicity) causes neuronal injury, while overactivation causes an increase in energy demand. By gradually raising the “powerA” parameter, one can model the increase in extracellular glutamate concentration caused by over-stimulation of NMDA receptors and exacerbation of excitotoxicity. The following numbers were used in the control model and the strength of the excitotoxicity phenomenon, respectively, from 9 to 135.

### 2.4. Therapy with the NMDA Antagonist Memantine

Memantine inhibits the NMDA receptor and avoids excitotoxicity in cases of prolonged pathological NMDA receptor activation and associated channel opening. As a result, memantine suppresses aberrant activation of the receptor without obstructing normal activation. Memantine improves the electrophysiological signal-to-noise ratio in this way. As a result, it is feasible to recognize a “meaningful” input, and physiological LTP, which is responsible for learning and memory, can be reestablished [37,38]. Studies [39,40,41,42,43] indicated that memantine inhibits NMDA receptor currents in a concentration-dependent manner, with IC50 values (concentration causing 50% inhibition) in the range 0.5–10 µM at hyperpolarized membrane potentials (−30 to −70 mV).

### 2.5. Parameters in Complex System—Neurodegenerative Disease

The reconstruction of the phase space as a way to characterize the complexity of the dynamic system was made possible by nonlinear analysis of the findings of the control model simulation of diseases [44].

The time delay approach was utilized to reconstruct the attractor [45,46]. The approach of false nearest neighbors, on the other hand, chose the smallest dimensions for deposition of a one-dimensional time series of neural network simulation outcomes [47]. The next step was to use Webber and Zbilut’s approach of recurrence quantification analysis to calculate correlation dimensions, Shannon entropy, and the positive maximal Lyapunov exponent [47]. The Shannon entropy of the simulation time series was calculated using the theory of communication of Shannon [48].

### 2.6. Parameters of Information Theory

Mutual information was recognized as an alternative to the correlation analysis in terms of the information theory [49,50,51]. The approach of entropy transfer was employed instead because mutual information measures how much information we can have about signal A knowing B, but does not provide knowledge about the dynamics and direction of flow [18,19].

### 2.7. Parameters of Synaptic Transmission

In simulations comparing control, AD, and memantine treatment models, the following parameters of synaptic transmission were used: number of spikes, LTP, and LTP time.

### 2.8. Statistical Methods and Software

TIBCO Software, Inc. (2017), Statistica (data analysis software system), version 13, (Palo Alto, CA, USA, 2017, http://statistica.io, accessed on 1 October 2021) was used for the statistical analysis. Fisher or Kruskal–Wallis tests were used to determine the significance of differences between more than two groups. Post hoc tests were done when there were statistically significant differences between two groups. For qualitative variables, chi-squared tests for independence were utilized. A correlation study was done to determine the Pearson or Spearman correlation coefficients in order to determine dependence, strength, and direction between variables. A statistical significance level α = 0.05 was utilized in all calculations. The Neuroscience Information Theory Toolbox program was used to conduct parameter calculations for complex systems and information theory [19].

## 3. Results

### 3.1. Parameters in Complex System—Neurodegenerative Disease

The mean values and 95% CI for Shannon entropy in AD and memantine treatment models were 1.760 (95% CI, 1.704–1.818) vs. 2.385 (95% CI, 2.280–2.490). There were statistically significant differences in Shannon entropy between AD and memantine treatment models (*p* = 0.0162). The test showed that Shannon entropy was significantly higher in the memantine treatment model relative to the AD model. The mean values and 95% CI for the positive Lyapunov exponent in the AD and memantine treatment models were 0.125 (95% CI, NE–NE) vs. 0.058 (95% CI, 0.044–0.073). There were statistically significant differences in the positive Lyapunov exponent between AD and memantine treatment models (*p* = 0.0091). The test showed that the positive Lyapunov exponent was significantly higher in the AD model relative to the memantine treatment model. The mean values and 95% CI for Lyapunov time in AD and memantine treatment models were 8 (95% CI, NE–NE) vs. 19 (95% CI, 14.8–22.5). The test showed that Lyapunov time was significantly higher in the memantine treatment model relative to the AD model (*p* = 0.0162) (Table 1).

The mean values and 95% CI for time delay in AD and memantine treatment models were 1.33 (95% CI, −0.10–2.77) vs. 3.33 (95% CI, 1.95–4.72), respectively. There was no statistically significant difference in time delay of the AD model and the treatment model (*p* = 0.1956). The mean values and 95% CI for the minimum embedding dimension in the AD and memantine treatment models were 2.67 (95% CI, −2.50–7.84) vs. 1.33 (95% CI, 0.95–1.72). There was no statistically significant difference in the minimum embedding dimension in the AD model and the treatment model (*p* = 0.3092). The mean values and 95% CI for correlation dimensions in the AD and memantine treatment models were 1.97 (95% CI, −3.37–7.32) vs. 3.98 (95% CI, −0.47–8.44). There was no statistically significant difference in the correlation dimension of the AD model and the treatment model (*p* = 0.4595).

### 3.2. Parameters of Information Theory

The mean values and 95% CI for transfer entropy in the AD and memantine treatment models were 0.081 (95% CI, 0.048–0.114) vs. 0.040 (95% CI, 0.019–0.062). There were statistically significant differences in transfer entropy between the AD and memantine treatment models (*p* = 0.0146). The test showed that transfer entropy was significantly higher in the AD model relative to the memantine treatment model.

**Figure 2:**
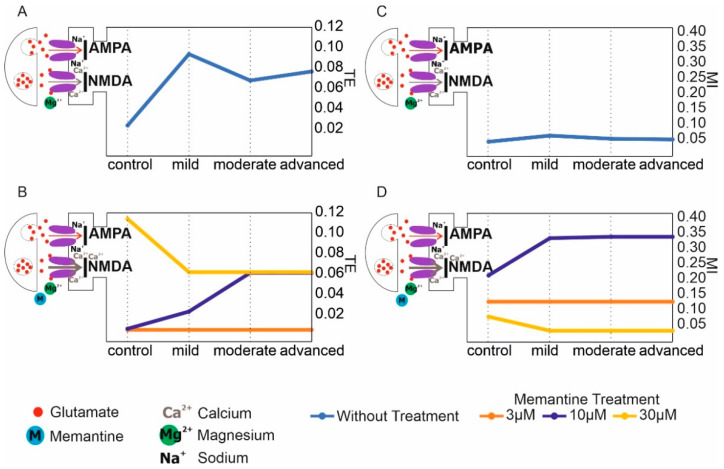
Synaptic transmission “as a function” of transfer entropy (TE) and mutual information (MI) in control, AD, memantine treatment models. (**A**) Transfer entropy of control and AD models. (**B**) Mutual information of control and AD models. (**C**) Transfer entropy of control and memantine treatment models at three concentrations: 3, 10, and 30 µM. (**D**) Mutual information of control and memantine treatment models at three concentrations: 3, 10, and 30 µM.

The mean values and 95% CI for mutual information in the AD and memantine treatment models were 0.061 (95% CI, 0.044–0.077) vs. 0.171 (95% CI, 0.067–0.275). There was no statistically significant difference in mutual information in the AD model and the treatment model (*p* = 0.4523) (Figure 2).

### 3.3. Parameters of Synaptic Transmission

The mean values and 95% CI for spikes in the AD and memantine treatment models were 134.0 (95% CI, 96.5–171.5) vs. 180.0 (95% CI, 154.2–205.8). The statistical test showed that the number of spikes was significantly higher in the memantine treatment model relative to the AD model (*p* = 0.0487). The mean values and 95% CI for LTP in the AD and memantine treatment models were 1.80 (95% CI, 1.76–1.84) vs. 1.92 (95% CI, 1.66–2.14). There were statistically significant differences in LTP between the AD and memantine treatment models (*p* = 0.0002). The test showed that LTP was significantly higher in the memantine treatment model relative to the AD model. The mean values and 95% CI for LTP time in the AD and memantine treatment models were 3.1 (95% CI, 1.7–4.4) vs. 5.4 (95% CI, −0.8–11.7). There were statistically significant differences in LTP time between the AD and memantine treatment models (*p* = 0.0465). The test showed that LTP time was significantly higher in the memantine treatment model relative to the AD model (see Supplementary Materials).

### 3.4. Relationships between Memantine Concentrations and Parameters in Complex System

A correlation analysis showed positive and statistically significant correlations of the memantine concentrations and minimum embedding dimension (correlation coefficient R = 0.87, *p* = 0.0025), correlation dimension (correlation coefficient R = 0.97, *p* < 0.00001), and positive Lyapunov exponent (correlation coefficient R = 0.87, *p* = 0.0023). Whereas there were statistically significant negative correlations of the memantine concentrations and Lyapunov time (correlation coefficient R = −0.87, *p* = 0.0023). In contrast, there was no statistically significant relationship between memantine concentrations and Shannon entropy (*p* = 0.1840) (Table 2).

### 3.5. Relationships between Memantine Concentrations and Parameters of Information Theory, Synaptic Transmission

The correlation analysis showed positive and statistically significant correlations of the memantine concentrations and TE (correlation coefficient R = 0.99, *p* < 0.000001). Whereas there were statistically significant negative correlations of the memantine concentrations and the number of spikes (correlation coefficient R = −0.99, *p* < 0.000001), LTP (correlation coefficient R = −0.87, *p* = 0.0025), and LTP time (correlation coefficient R = −0.85, *p* = 0.0037). In contrast, there was no statistically significant relationship between memantine concentrations and MI (*p* = 0.1773) (Table 3).

## 4. Discussion

Memantine, when given in amounts that have neuroprotective effects, was also found to improve memory in several trials. The biological findings are confirmed by our computer simulations. Our findings are in line with a slew of preclinical research that demonstrate therapeutic doses of memantine exhibit neuroprotective properties. They do not cause side effects in learning impairment and long-term synaptic potentiation. Our study also confirms that, as in experiments in healthy volunteers, memantine at higher doses did not affect cognition [52,53,54].

Experiments suggest that memantine inhibits pathological alterations in the hippocampus [11], and that it prevents neuronal death in rats when given before NMDA injections [55]. We corroborate the usage of memantine in models of excitotoxicity severity in our simulation tests (mild, moderate, and advanced) resulted in shortened Lyapunov time, number of spikes, LTP, LTP time, increased minimum embedding dimension, correlation dimension, positive Lyapunov exponent, and transfer entropy. The increased value of Shannon entropy in AD vs. the control model was shown in previous studies [23,24,25].

The calculated Lyapunov exponents showed that the control, AD, and memantine treatment models had positive values (i.e., they were unstable systems with chaos). The positive Lyapunov exponent was higher in the control model versus the AD and memantine treatment model. In the memantine treatment model, the positive Lyapunov exponent was close to zero, i.e., the system was at its most stable stage of development. The calculated Shannon entropy values showed its increase from AD to the memantine treatment model. It is the law of increasing entropy, which is general in nature and applies to all processes in nature. The increased value of Lyapunov time in the memantine treatment model was shown in our study, compared to the control and AD models.

Simulation studies suggest that generating gamma oscillations in the hippocampus may help to minimize Alzheimer’s disease pathogenesis. After gamma induction, pathologically elevated transfer entropy values reverted to levels equivalent to the control model [31]. In our study, the use of memantine therapy at a dose of 10 µM in Alzheimer’s patients causes a pathological reduction in entropy transfer and returned to values comparable to the control model.

Some studies showed no statistically significant differences in cognitive function versus placebo [56,57,58,59,60]. The mean cognitive function scores for patients with Alzheimer’s disease and the control group were −4.10 to 2.41 and −2.80 to 5.60, respectively. Memantine monotherapy improved cognitive function scores in patients with Alzheimer’s disease from −0.80 to 4.00 vs. 1.10 to 10.10 in four studies compared to the placebo.

The results of the mutual information analysis showed a very strong linkage between the arrival of an action potential and postsynaptic potential of memantine treatment at a 10 µM concentration, while in the interaction of an action potential and postsynaptic potential in the control, pathological models were weak. However, the calculated Shannon information entropy values showed its decline with increasing memantine concentrations, while transfer entropy increased. Whereas, for the same process, MI, LTP, LTP time, and the number of spikes, decreased.

The analysis of simulation studies from the pyramidal cell to the hippocampal network in information theory shows the trajectory in a phase space converges up to a (nearly) point attractor [26] and there was a decrease in entropy, which was caused by an increase of the forgetting coefficient (numerical parameter, their changes allow confining the strength and time of LTP to biologically plausible values for any kind of modeled neuron) in pyramidal cells [28]. A synaptic breakdown with an increase in Shannon entropy implies an Alzheimer’s disease phase that is irreversible. Increased synapse loss resulted in decreased information flow and entropy transfer in DG → CA3, but a significant increase in CA3 → CA1, at the same time [29]. Simulation studies suggest that generating gamma oscillations in the hippocampus may help to minimize Alzheimer’s disease pathogenesis. After gamma induction, pathologically elevated transfer entropy values reverted to levels equivalent to the control model [31].

## 5. Conclusions

The information theory results of simulation studies show that the NMDA antagonist, memantine, causes neuroprotective benefits in patients with AD. Our simulation study opens up remarkable new scenarios in which a medical product, drug, or device, can be developed and tested for efficacy based on parameters of information theory.

## 6. Future Directions

In future studies, we would like to attempt an in silico study comparing virtual DBS therapy with NMDA antagonist treatment, including deep brain stimulation (DBS) in AD patients. Computer modeling and in silico studies are successfully used to reduce, refine, and partially replace animal and human experiments; in particular, the use of induced gamma oscillations in the hippocampus ameliorates the pathology associated with Alzheimer’s disease.

## 7. Limitations

Any simulation study that relies on a mathematical and computer model is a restriction of the investigation. Despite the fact that our model accurately captures the mechanics of the NMDA receptor function and excitotoxicity severity simulation, it will never be able to match the complexity of biological systems.

## Figures and Tables

**Table 1:** Parameters in the complex system—neurodegenerative disease: Shannon entropy, positive Lyapunov exponent, and Lyapunov time in control, AD, memantine treatment models.

Group	Shannon Entropy	Positive Lyapunov Exponent	Lyapunov Time
**Control model**	1.111	0.200	5
**AD model**	1.760 ^1^	0.125 ^1^	8 ^1^
mild	1.773	0.125	8
moderate	1.734	0.125	8
advanced	1.773	0.125	8
**Memantine treatment model**	2.385 ^2^	0.058 ^2^	19 ^2^
3 µM	2.333	0.045	22
10 µM	2.560	0.045	22
30 µM	2.261	0.083	12

^1^ Mean of three stages AD, ^2^ mean of three concentrations of memantine.

**Table 2:** Correlation analysis of minimum embedding dimension, correlation dimension, Shannon entropy, positive Lyapunov exponent, and Lyapunov time in memantine treatment models.

Parameters	R ^1^	*p*-Value
Minimum embedding dimension	0.87	0.0025
Correlation dimension	0.97	<0.00001
Shannon entropy	−0.49	0.1840
Positive Lyapunov exponent	0.87	0.0023
Lyapunov time	−0.87	0.0023

^1^ Spearman’s correlation coefficient.

**Table 3:** Correlation analysis of transfer entropy (TE), mutual information (MI), spikes, LTP, and LTP time in memantine treatment models.

Parameters	R ^1^	*p*-Value
TE	0.99	<0.000001
MI	−0.49	0.1773
Spikes	−0.99	<0.000001
LTP	−0.87	0.0025
LTP time	-0.85	0.0037

^1^ Spearman’s correlation coefficient.

## Data Availability

All data generated or analyzed during this study are included in this published article (and its Supplementary Materials files).

## Supplementary Materials

The following supporting information can be downloaded at: https://www.mdpi.com/article/10.3390/ijerph19084727/s1.
